# Fast responses to images of animate and inanimate objects in the nonhuman primate amygdala

**DOI:** 10.1038/s41598-020-71885-z

**Published:** 2020-09-11

**Authors:** E. Cleeren, I. D. Popivanov, W. Van Paesschen, Peter Janssen

**Affiliations:** 1 Laboratory for Neuro- and Psychophysiology, and the Leuven Brain Institute, KU Leuven, Herestraat 49 bus 1021, 3000 Leuven, Belgium; 2 Laboratory for Epilepsy Research, KU Leuven, Leuven, Belgium; 3 Department of Cognitive Science and Psychology, New Bulgarian University, Sofia, Bulgaria; 4 Medical University of Sofia, University Hospital “Alexandrovska”, Sofia, Bulgaria

**Keywords:** Neuroscience, Cognitive neuroscience, Neuronal physiology, Visual system

## Abstract

Visual information reaches the amygdala through the various stages of the ventral visual stream. There is, however, evidence that a fast subcortical pathway for the processing of emotional visual input exists. To explore the presence of this pathway in primates, we recorded local field potentials in the amygdala of four rhesus monkeys during a passive fixation task showing images of ten object categories. Additionally, in one of the monkeys we also obtained multi-unit spiking activity during the same task. We observed remarkably fast medium and high gamma responses in the amygdala of the four monkeys. These responses were selective for the different stimulus categories, showed within-category selectivity, and peaked as early as 60 ms after stimulus onset. Multi-unit responses in the amygdala were lagging the gamma responses by about 40 ms. Thus, these observations add further evidence that selective visual information reaches the amygdala of nonhuman primates through a very fast route.

## Introduction

For many decades, a large number of studies has investigated the unconscious processing of emotions (reviewed in Adolphs^1^ and Tamietto^2^). During backward masking or binocular rivalry, non-consciously perceived emotional stimuli can elicit physiological responses associated with autonomic arousal, and neuroimaging studies have consistently shown activations in a number of subcortical structures caused by non-consciously perceived emotional stimuli, such as the amygdala, superior colliculus, pulvinar and basal ganglia. A widely proposed pathway through which visual information could reach the amygdala is via the superior colliculus and the pulvinar, bypassing the ‘conscious’ ventral visual pathway. However, this ‘low road’ pathway is highly debated (see Morris^3^, Pessoa^4^ and Silverstein^5^).

In nonhuman primates, the amygdala receives highly processed information from the inferior temporal cortex (ITC) through the perirhinal cortex^6,7^. More recently, Grimaldi, Saleem and Tsao^8^ showed that the so-called face patches, which contain a high concentration of neurons responding more to faces than to objects, are also strongly connected to the amygdala.

Mendez-Bertolo et al.^9^ measured very fast evoked potentials elicited by fearful faces in the amygdala of humans, providing the first evidence for a fast processing pathway for emotional information in primates. Mc Fadyen et al.^10^ provided evidence based on magnetoencephalography (MEG) that supports a subcortical pathway in humans, in which visual information arrives at the amygdala as early as 70 ms, regardless of emotional expression or spatial frequency. However, a number of questions still remain. Firstly, it is unclear whether similarly fast electrophysiological responses can be detected in the amygdala of nonhuman primates, which represent the main animal model for object recognition in humans. Secondly, because the origin of the evoked potential and the source of the MEG signals are unclear, it remains to be determined whether other electrophysiological measurements that correlate more closely with population activity, such as the gamma frequency band of the local field potential (LFP), would also respond at very short latencies. Finally, we need further evidence whether ultra-fast amygdala responses can also be evoked by presenting images of neutral faces, bodies or objects, without emotional content.

We recorded in the amygdala of four rhesus monkeys performing a passive fixation task, while images of different object categories appeared on a display, and analyzed the gamma band responses of the LFP. Both the medium (50–80 Hz) and the high (80–150 Hz) gamma activity emerged shortly after stimulus onset, for all stimulus categories, and peaked around 60 ms after stimulus onset. Surprisingly, these ultra-fast gamma responses showed significant selectivity between stimulus categories and even within stimulus categories. In contrast, multi-unit responses in the amygdala were lagging the gamma responses by at least 40 ms. Thus, these observations represent the first evidence of ultra-fast visual processing in the amygdala of nonhuman primates.

## Results

**Figure 1:**
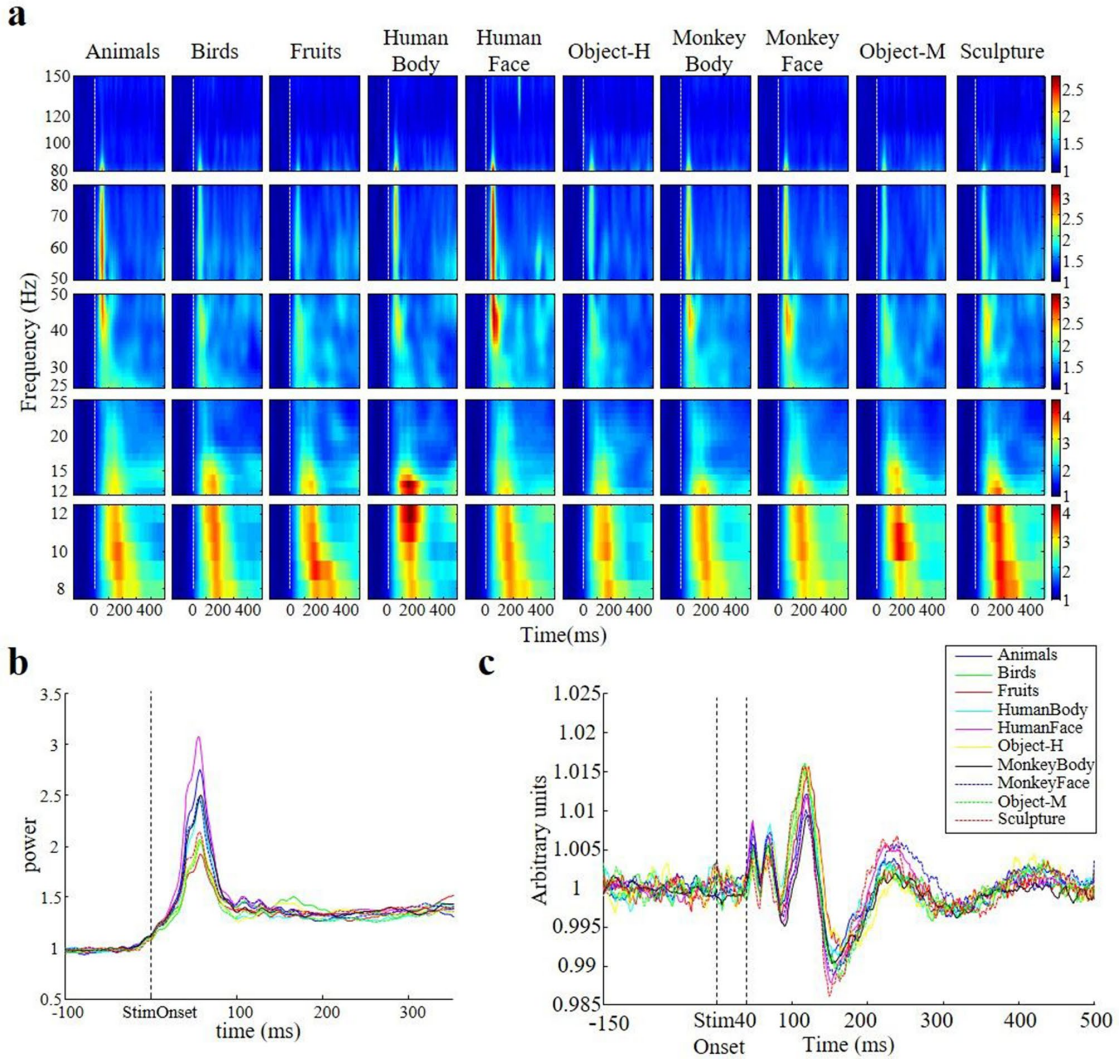
Category selectivity of the LFP power. (**a**) Time–frequency plots representing the power change normalized to baseline for one example site of monkey K. for each of the 10 stimulus classes, subdivided into different frequency bands. Stimulus onset is marked by a white vertical dotted line. (**b**) For the same example site, we plotted the average power in the medium gamma band (50–80 Hz) as a function of time. (**c**) The visually-evoked potential (VEP, calculated as the mean LFP signal aligned on stimulus onset) of the same example site. The onset of the VEP was at 40 ms after stimulus onset.

We recorded LFPs in 58 sites (15 with chronically implanted electrodes and 43 using acute electrodes) in the amygdala of four monkeys. The time–frequency plot of an example site recorded with a chronically implanted electrode in monkey K. is illustrated in Fig. 1a (N = 14,379 trials obtained in 10 sessions). All stimulus categories elicited strong LFP responses in the alpha and beta bands that were not category-selective (Anova, p = 0.07 and 0.55 in the 30–100 ms time window for alpha and beta band, respectively). However, in the gamma band we measured highly category-selective and remarkably fast LFP responses. This response pattern was most conspicuous for the medium gamma band (50–80 Hz): in this frequency band, human faces evoked a strong (+ 200% increase), transient and category-selective (p = 1.18e−22, Anova on the interval 30–100 ms after stimulus onset) response that peaked as early as 58 ms after stimulus onset.

To better appreciate the temporal dynamics of the gamma responses in the amygdala, we plotted the average power in the medium gamma band as a function of time for the different stimulus categories (Fig. 1b). Every stimulus category evoked a significant but transient medium gamma response, with a maximum ranging between + 70 and + 200% at 58 ms after stimulus onset, followed by a rapid decay within 100 ms to a steady-state level of approximately + 35% compared to baseline. Importantly, the medium gamma response was highly category-selective in the interval 30–100 ms after stimulus onset (Anova, p = 1.18e−22, with similar results in the interval 0–200 ms), but did not show any selectivity in the later part of the response (Anova on the interval 200–500 ms after stimulus onset, p = 0.84). Similarly, the onset of the visual-evoked potential was as early as 40 ms after stimulus onset (Fig. 1c).

**Figure 2:**
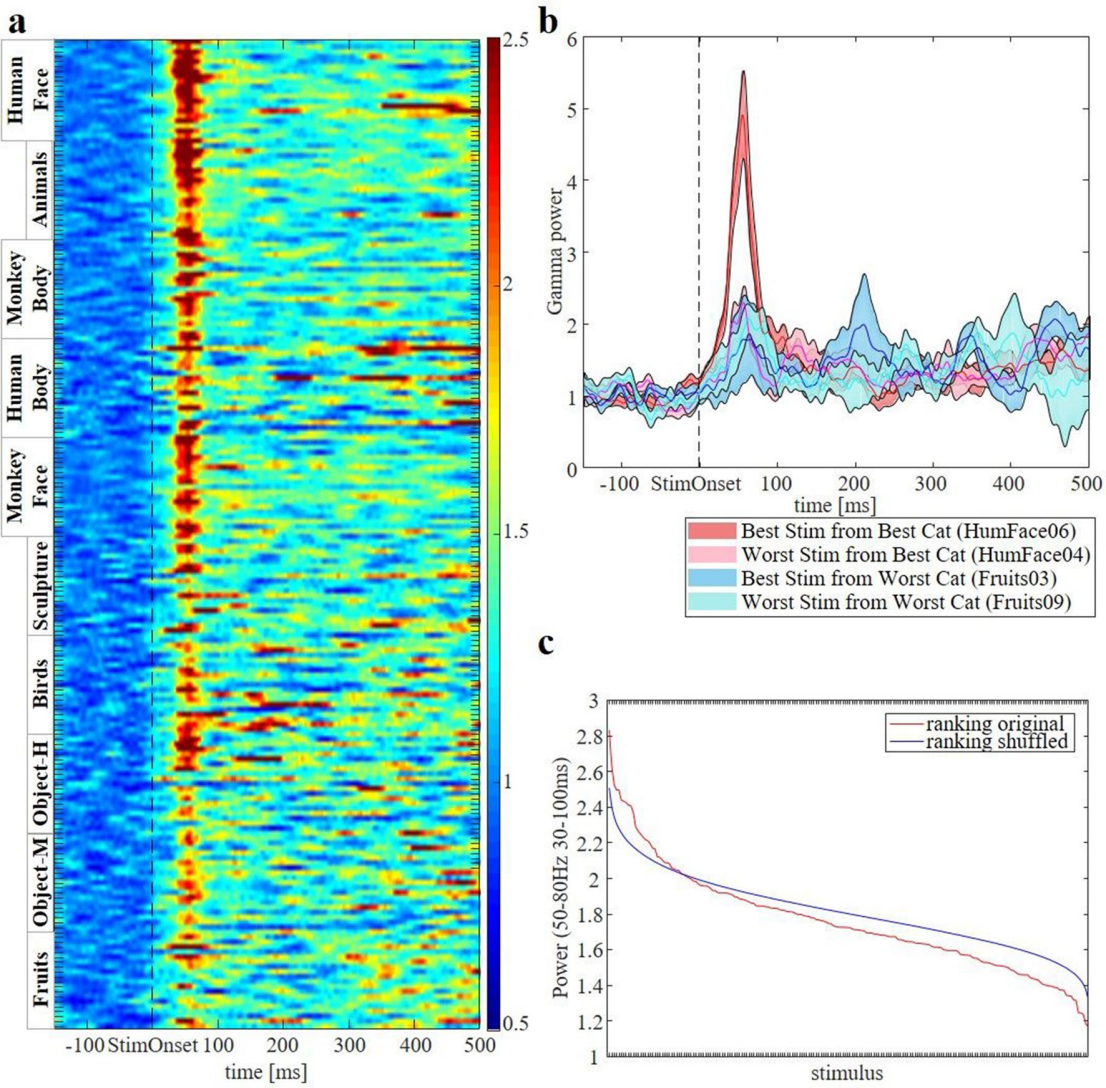
LFP selectivity for individual stimuli of the same example site as Fig. 1. (**a**) Medium gamma time frequency plot ranked across stimuli category and within categories based on the ranking of the other half of the recorded trials. This plot shows a marked ranking across and within categories. (**b**) Power time plot of the best and worst stimulus in the best and worst category. (**c**) Medium gamma power in function of ranked stimulus (red curve). Randomly shuffling the stimulus numbers (blue curve) yields a ranking nonparallel to the original ranking, further demonstrating the category selectivity.

Since we obtained a very large number of trials (N = 14,379) in this recording site, we could also investigate the selectivity of the medium gamma response for each of the 200 individual images. In Fig. 2a, we first ranked the average medium gamma responses using the odd trials across stimulus category, then ranked the individual images of each category (in the interval 30–100 ms after stimulus onset), and plotted the even trials according to this ranking. As expected, most images of the preferred category (human faces) evoked strong medium gamma responses, whereas the least effective category (fruits) contained much less images evoking strong medium gamma responses. However, Fig. 2a clearly demonstrates that we measured a wide range of responses within each category. To further demonstrate the robust within-category selectivity in this recording site, Fig. 2b shows the average medium gamma responses to the best and worst images of the most effective and the least effective categories. In this example recording site, human face 06 elicited 2.1 times more medium gamma response than human face 04 in the interval from 30 to 100 ms after stimulus onset. Thus, the medium gamma response in the amygdala shows strong between-category and within-category selectivity at remarkably short latencies. Additionally, Fig. 2c illustrates that, by randomly shuffling the stimuli to the trials 1,000 times, we lose the original ranking, giving further evidence of the neural selectivity, even at the within-category level.

The example recording site in Figs. 1 and 2 clearly demonstrates that we observed robust category selectivity in the medium gamma response recorded in the amygdala. Half of the chronic recording sites (7/15) and 4/43 acute recording sites showed significant differences in the medium gamma response between the ten stimulus categories tested (Anova, 30–100 ms after stimulus onset, p < 0.05). The preferred stimulus category differed between recording sites: in four recording sites, sculptures were the preferred stimulus category, human faces in three recording sites, and fruits, monkey bodies, human bodies or animals were the preferred stimulus category in one recording site. Hence the presence of a (human or monkey) face did not always result in the highest medium gamma response in the amygdala.

**Figure 3:**
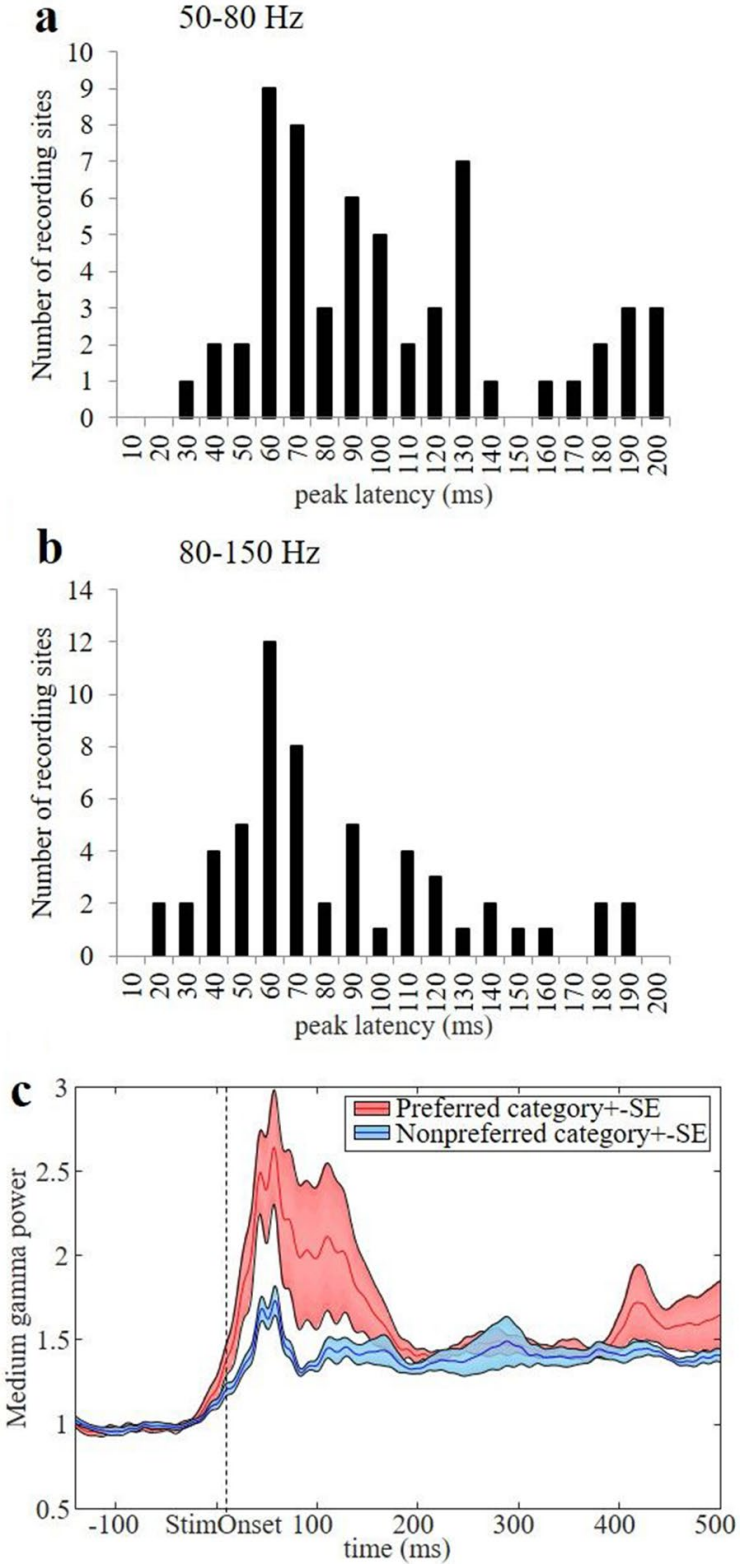
Distribution of the peak latency of the preferred stimulus category across all LFP sites in the medium gamma (**a**) and high gamma (**b**) band. (**c**) Medium gamma power of the preferred and nonpreferred category across the category-selective sites (n = 9, ANOVA 0–200 ms, p < 0.05).

For each recording site, we determined the time at which the average medium and high gamma response to the preferred stimulus category reached a maximum in the interval from 0 to 200 ms after stimulus onset (to exclude secondary peaks in the gamma response occurring later in time). The distribution of these peak times is shown in Fig. 3a,b. In more than half of the recording sites (36/58, 62%), the medium gamma response to the preferred stimulus category peaked earlier than 100 ms after stimulus onset (41/58 or 71% for the high gamma response; median peak time = 87 ms for medium gamma and 68 ms for high gamma), and the mode of the distribution was located at 60 ms after stimulus onset. Intriguingly, the fastest gamma responses already peaked at 20–30 ms after stimulus onset. Furthermore, we frequently observed significant category selectivity within 100 ms after stimulus onset: 8 out of 36 (22%) of the recording sites with a medium gamma peak less than 100 ms after stimulus onset showed significant category selectivity (in the interval 0–200 ms after stimulus onset, Anova, p < 0.05), compared to 2 out of 41 sites (5%) with an early (< 100 ms) high gamma peak. Across all amygdala recording sites, significant category selectivity was not uncommon when tested with chronically implanted electrodes (7/15 or 47%), but much less frequent for the acute recordings (4/43 or 9%), most likely due to the smaller number of trials in the acute recordings (on average 647 trials per recording site, compared to 11,042 for the chronic recordings). To estimate at which point in time significant category information emerged in the medium gamma response in the amygdala, we averaged the medium gamma responses to the preferred and nonpreferred stimulus category across all category-selective recording (0–200 ms after stimulus onset, Anova, p < 0.05) sites. Category selectivity was clearly present around 50 ms after stimulus onset (Fig. 3c).

The medium gamma response in the amygdala was not only very early, but also remarkably strong. Across all recording sites, the peak of the medium gamma response in the interval 0–200 ms after stimulus onset ranged from + 46 to + 9,877% compared to baseline for the preferred stimulus, with a median average evoked medium gamma response of + 122% in that same interval.

**Figure 4:**
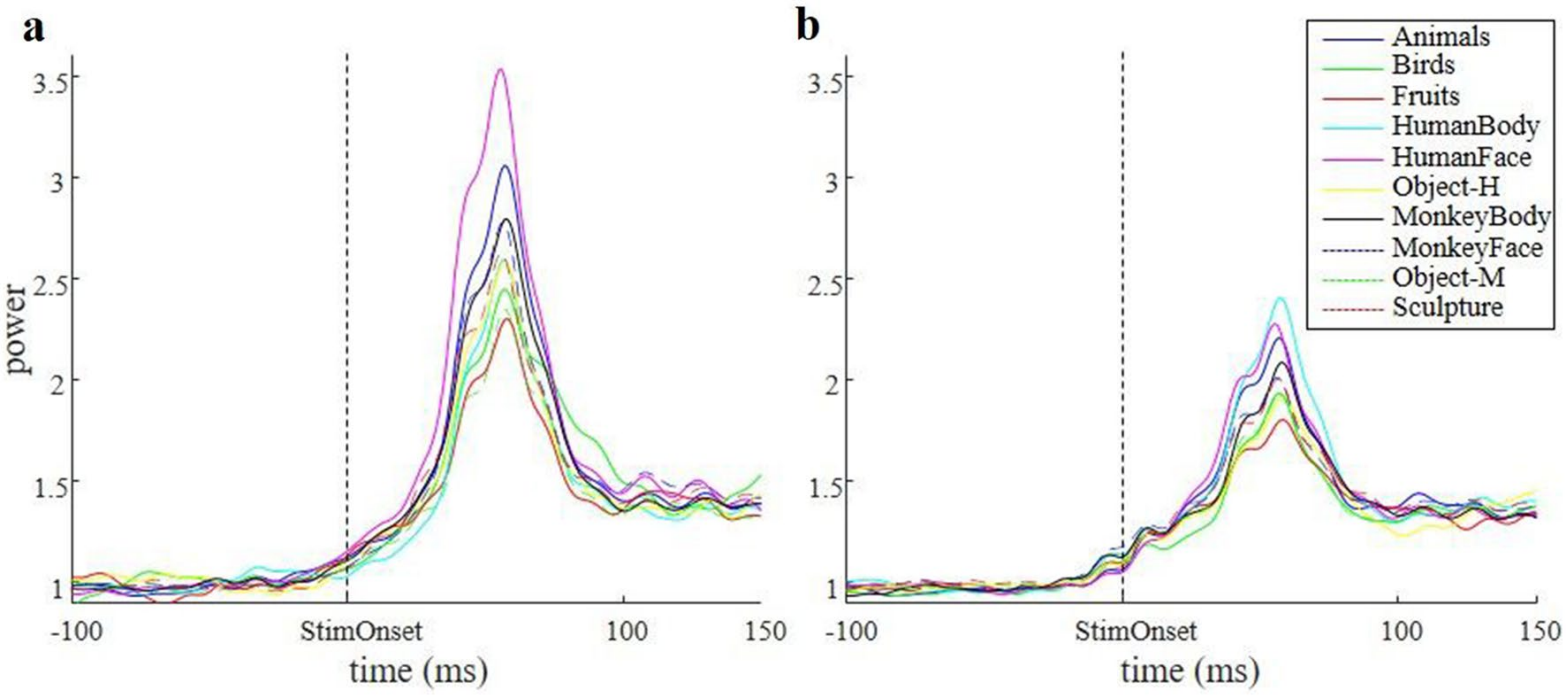
Average LFP power in the medium gamma band (50–80 Hz) over recording sessions. We split the full dataset of the example site in two parts, yielding respectively 6,569 (panel **a**, first half) and 8,001 (panel **b**, second half) correct trials. Anova in the time window of 30–100 ms was significant in both parts of the data (p = 2.1e−05 and 0.0081 for the first and second half respectively).

To examine whether the fast gamma responses in the amygdala reflected stimulus novelty, we divided the data set on the example electrode in two parts, and plotted the average 50–80 Hz gamma responses in the first 6,569 trials compared to the last 8,001 correct trials (Fig. 4a,b). Although the strength of the gamma responses was lower in the second part of the data set, we still measured selective (Anova, p = 0.008) and very fast (peak at 60 ms) medium gamma responses in this second part of the data set.

**Figure 5:**
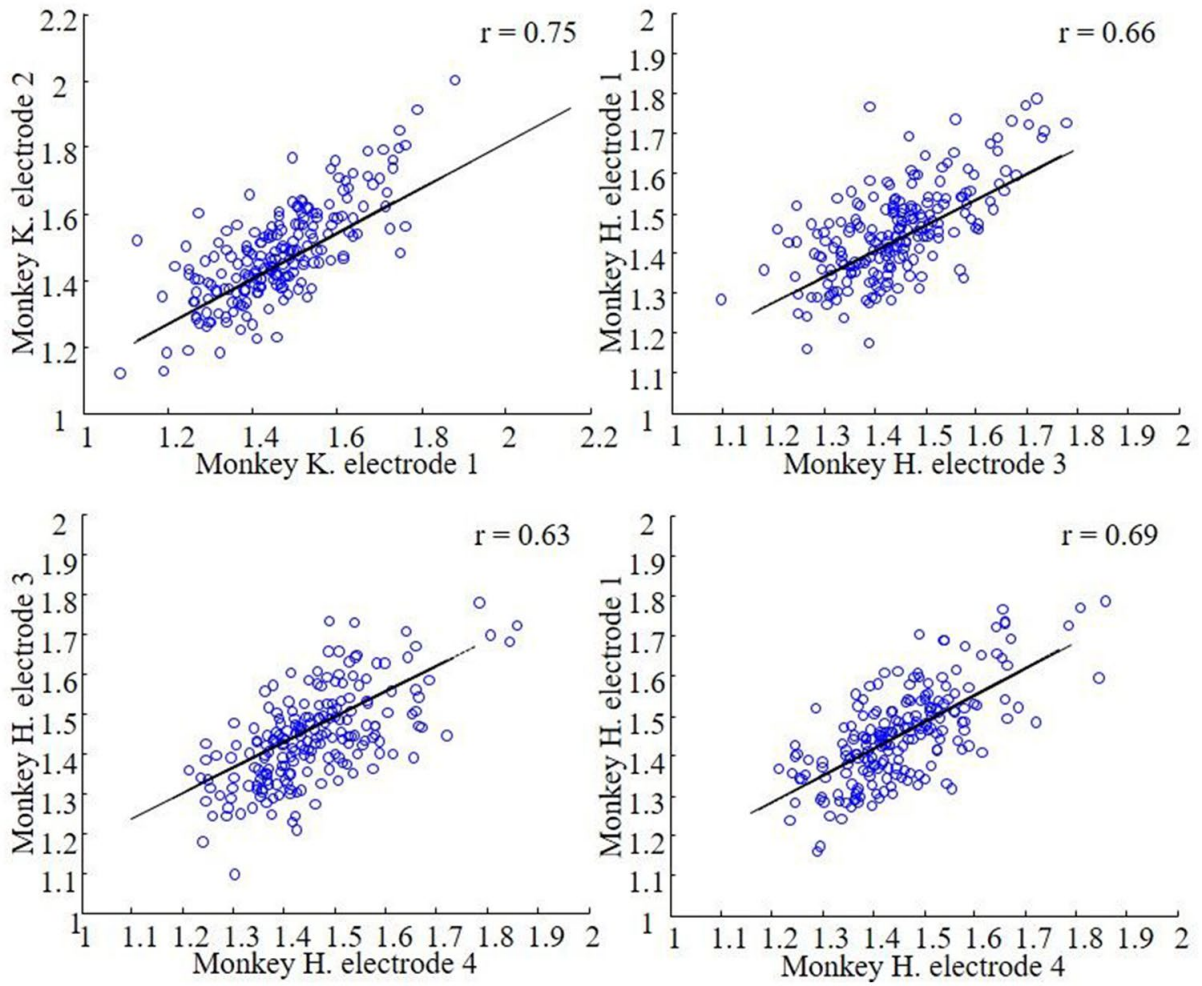
Correlations of the average medium gamma response to each stimulus on pairs of chronically implanted electrodes that showed a significant category selectivity. All correlations were significantly larger than zero, showing that the gamma responses we measured using the chronically implanted electrodes in the same subject were correlated but not identical.

We investigated whether the medium gamma responses to the 200 images in the stimulus set were similar on different (chronically implanted) electrodes, which were located approximately 1–2 mm apart. To that end, we correlated the average medium gamma responses to each image on pairs of electrodes showing significant stimulus selectivity (Anova p < 0.05). Figure 5 illustrates that the correlations between pairs of electrodes varied between 0.63 and 0.75 (all significantly larger than zero), indicating that the gamma responses we measured were correlated but not identical on different electrodes.

**Figure 6:**
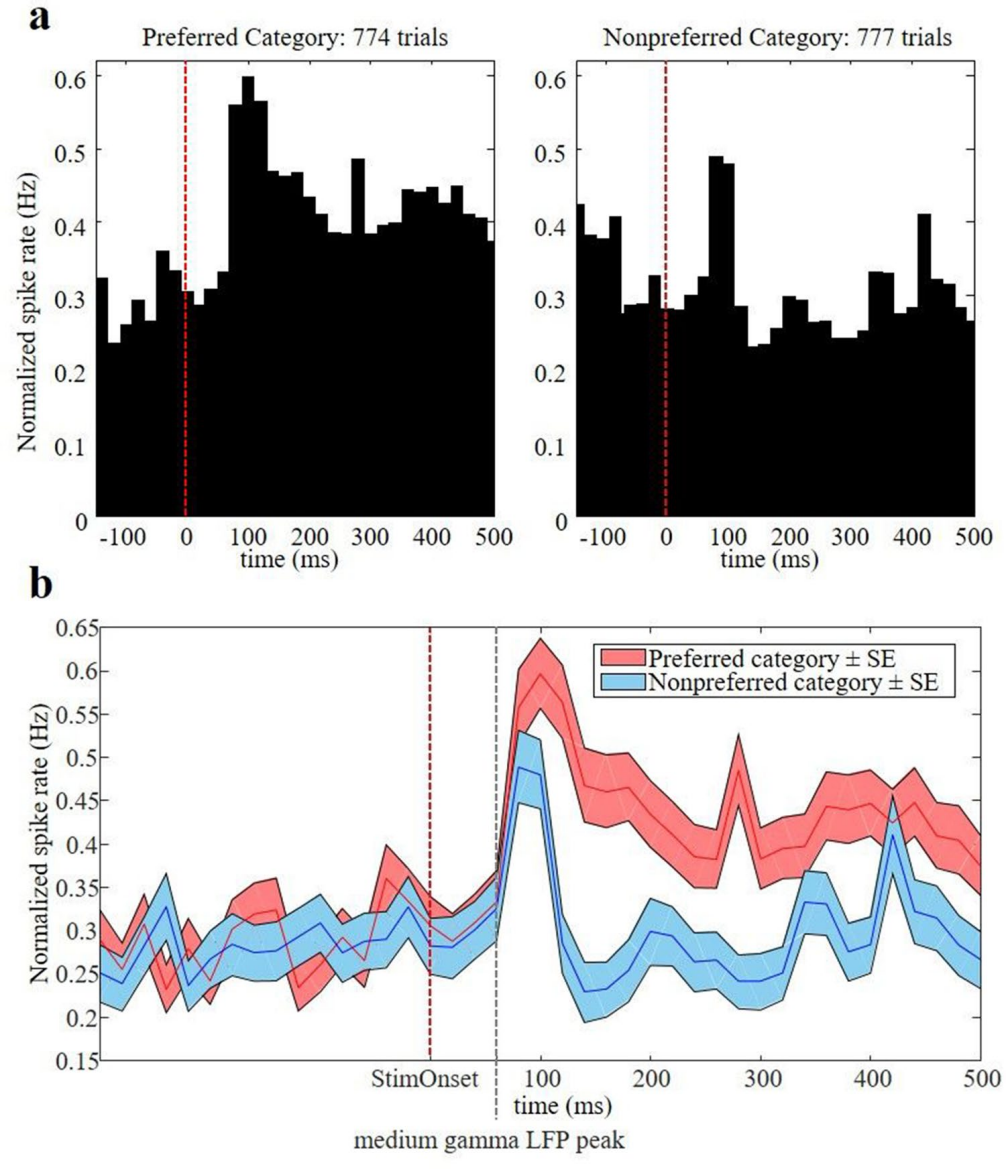
Multi-unit activity. The average multi-unit spiking response to the preferred and nonpreferred category. Red dashed line indicated stimulus onset. (**a**) Average spike histograms. (**b**) The average multi-unit spike rate ± SE. The grey dashed line indicates the mode of the peak value of the medium gamma power (60 ms), indicating that the peak information of the multi-unit response lags the peak information in the medium gamma response by about 40 ms.

Finally, we wanted to compare the medium gamma responses in the amygdala to the multi-unit spiking responses to the same images. In 20 out of 43 recording sites (monkey Sh), we measured visually responsive multi-unit activity. The average multi-unit spiking response to the preferred and nonpreferred stimulus category is illustrated in Fig. 6a,b. Although the response latency of the spiking activity was relatively short (70 ms, t-test p < 0.05 on three consecutive 20 ms bins after stimulus onset for the preferred category), the peak of the spiking response occurred around 100 ms after stimulus onset, and category selectivity (t-test on the response to the preferred category compared to the nonpreferred category) did not emerge before 100 ms after stimulus onset. Thus, information about the stimulus category in the multi-unit spiking response lagged category information in the medium gamma response by about 40 ms.

## Discussion

We observed exceedingly fast gamma responses (50–80 Hz and 80–150 Hz) in the amygdala of monkeys in response to images of faces, bodies, animals and objects. These ultra-fast gamma responses peaked as early as 60 ms after stimulus onset, and were selective for different stimulus categories and even within stimulus category.

A recent study^9^ reported fast amygdala evoked potentials in human patients in response to fearful (but not neutral) facial expressions. The intracranial event-related potentials (iERPs) in this study emerged at 74 ms after stimulus onset, which is slightly longer than in our study but comparable to our data given the larger size of the human brain (and hence longer conduction times). Several differences between our observations and Mendez-Bertolo et al.^9^ are important for the interpretation of the results. Firstly, we analyzed medium and high-gamma responses, whereas Mendez-Bertolo et al.^9^ reported evoked potentials. The evoked potential is dominated by the lower frequencies (theta, alpha and beta), which in our study were not category-selective. The gamma band of the LFP, on the other hand, correlates well with multi-unit spiking responses^11–13^. Therefore, our observations reflect much more the actual neuronal responses in the amygdala. In our study, the Visual Evoked Potential (VEP) started already around 40 ms after stimulus onset, but was not category-selective. Secondly, we only used neutral stimuli (images of human and monkey faces, bodies, animals and objects) whereas Mendez-Bertolo et al.^9^ observed fast iERPs for fearful faces but not for other stimulus categories. We could not determine to what extent some of our stimuli were arousing or threat-related, therefore the emotional content of the stimulus may have contributed to the observed selectivity of the fast gamma responses in the amygdala. Moreover, novelty was most likely not an important factor since our animals were extensively exposed the same stimuli prior to and during the recordings, and we measured very fast gamma responses even in the second half of the recordings (Fig. 4). These observations imply that the amygdala receives very fast information about stimulus category, which can be associated to an autonomic response after conditioning (e.g. for face stimuli paired with an unpleasant noise). However, the presence of fast category-selective responses in the amygdala does not imply that these responses constitute a neural representation of object categories, nor that they represent the basis for object categorization. Previous studies have demonstrated that categorization depends primarily on the prefrontal cortex^14^, in concert with visual information available in the inferotemporal cortex^15^ (for a potential role of the parietal cortex, see Zhou et al.^16^). Thirdly, probably because we measured the higher frequencies of the LFP, we even observed within-category selectivity. For example, the medium-gamma response was significantly different for two human faces as early as 60 ms after stimulus onset. This within-category selectivity in the medium gamma responses is much faster than what has been reported with identical stimuli in the inferior temporal cortex^17^. Note, however, that our monkey experiments most likely contained many more trials than the human recordings (which were acquired in a small number of sessions). Finally, we also measured multi-unit spiking activity, and could therefore confirm that the fast gamma responses preceded the single-unit responses in the amygdala by approximately 40 ms.

Although we could replicate our findings in every animal in our study (4 in total), the fast gamma selectivity was not equally frequent in every animal, and we only observed category selectivity in half of the recording sites tested with chronically implanted electrodes. Since the chronic recordings were obtained using a bundle of microelectrodes inserted in the amygdala, we had little control over the exact anatomical location of each electrode tip. Thus, the possibility remains that the early category selectivity in the gamma responses in the amygdala is only present in specific nuclei of the amygdala.

Kreiman et al.^18^ reported that the latency of the evoked potential (not the gamma band response) was 100 ms in the ITC, while in our amygdala data this was 40 ms (see Fig. 1c). Moreover, Popivanov et al.^17^ used the same stimulus set as in our study in the ITC of monkeys, and observed that the peak of the medium and high gamma responses in the ITC was 100 ms, while in our amygdala data most recording sites peaked at or before 60 ms after stimulus onset. Given that the amygdala is at least one synapse away from the anterior ITC (which amounts to a 10 ms delay), all recording sites that peaked before 100 ms must have bypassed the ITC (62% for medium gamma and 71% for high gamma). Mormann et al.^19^ presented an overview of a large number of studies investigating the latencies of the spiking responses—not the LFP responses—in the ITC, albeit with different stimulus sets (which makes it difficult to compare because the luminance can affect the latency). Nevertheless, almost all of these studies reported latencies not shorter than 80 ms, which could theoretically have an effect in the amygdala not earlier than 90 ms after stimulus onset. For the above reasons, it is clear that a very large part of the amygdala responses we measured occurred earlier than in the ITC, which can only be explained by another route bypassing the ITC.

The dissociation we observed between the early onset of the medium and high-gamma responses and the longer latency of the spiking responses is intriguing. At the very least, the fast and selective gamma responses we observed suggest that category-selective visual information reaches the amygdala through a very fast route bypassing the ITC, since both the spiking and the gamma responses in the ITC show much longer latencies^17^. Under the assumption that the medium and high gamma band largely reflect the inputs to the amygdala, our data imply that around 40–60 ms after stimulus onset, highly processed inputs arrive in the amygdala signaling the stimulus category—and possibly orchestrating the emotional response after conditioning—before the larger (pyramidal) neurons start to respond. A similar dissociation between gamma and single-unit responses has been observed in area LIP^13^. Thus, the fMRI activations in the amygdala evoked by emotional stimuli that were not consciously perceived may also largely reflect inputs to and local processing in the amygdala, rather than the (spiking) output of amygdala neurons. These inputs must then also evoke the autonomic responses associated with emotional stimuli.

Our results may represent the first electrophysiological evidence for a fast and unconscious route of visual information to the amygdala in nonhuman primates. The main advantage of the nonhuman primate model is that it now becomes possible to test specific predictions about the origin of these inputs. For example, if the pulvinar is indeed a major source of the fast gamma responses in the amygdala, we would expect that reversible inactivation of the pulvinar (using muscimol) would markedly reduce these fast gamma responses. We would also expect a change in the strength of the fast gamma responses for a specific category after conditioning the animal with stimuli from this category. Finally, future studies should also investigate whether similar category-selective fast gamma responses and slower spiking responses exist in the human amygdala. If so, both invasive^20^ and noninvasive^21^ neuromodulation interventions may become helpful to precisely shape the amygdala responses in patients with anxiety disorders.

## Materials and methods

### Subjects

**Figure 7:**
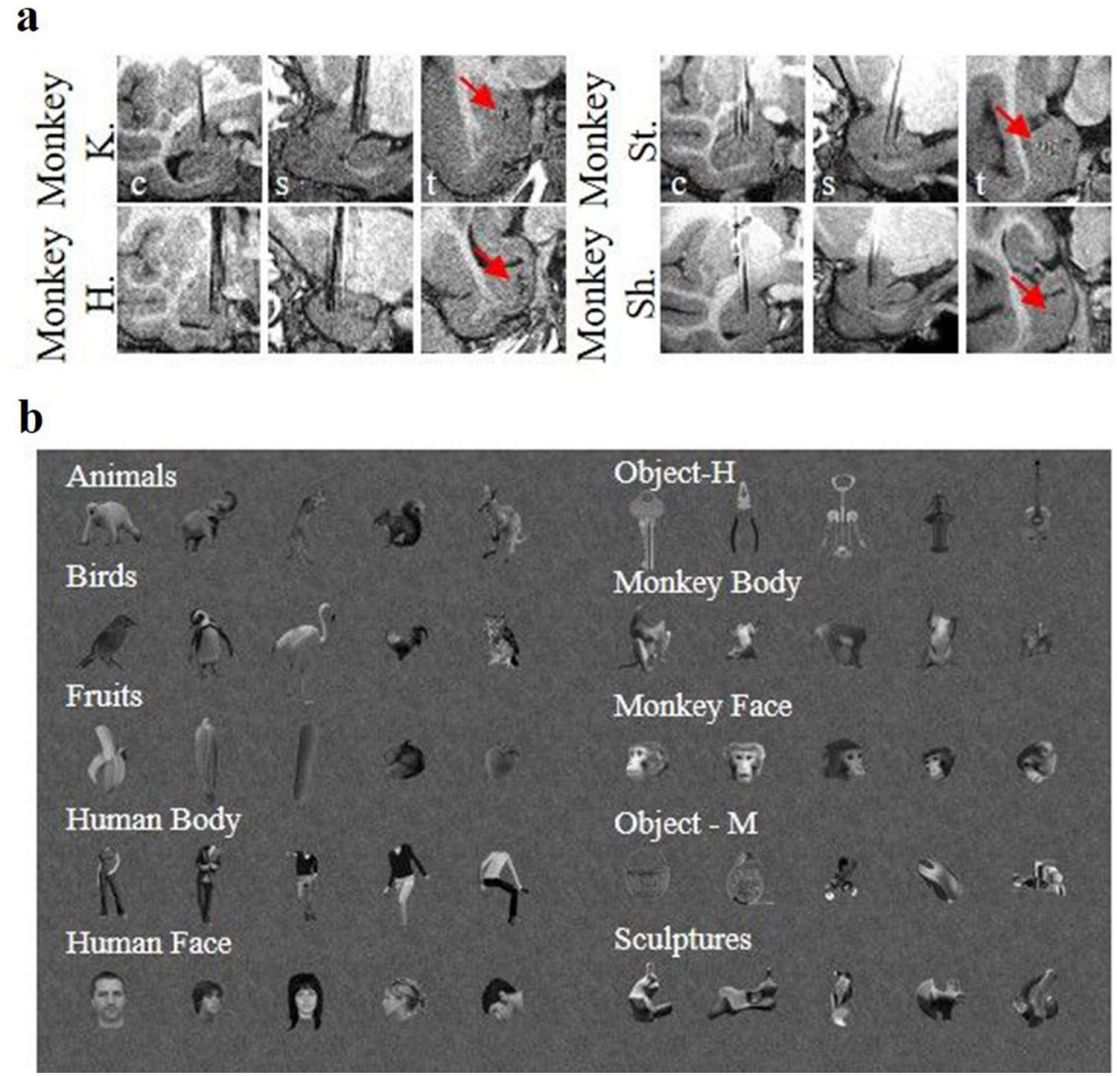
(**a**) Electrode position in each of the subjects verified with a T1-weighted MRI scan (*c* coronal view, *s* sagittal view, *t* transversal view). Note that the MRI of monkey Sh was left–right flipped. (**b**) Examples of the ten categories of visual stimuli used. The source and characteristics of the stimuli are described in great detail in Popivanov et al.^17^.

Four male rhesus monkeys (Macacca mulatta, weight 6–9 kg) participated in the present study. They were implanted with a headpost (Crist Instruments) for fixing the head during training and recording. Next, three of them were implanted with several (3 in monkey K., 5 in monkey H., 7 in monkey St.) long, MR compatible, tungsten electrodes (125 µm diameter, average impedance at 1 kHz: 130 kΩ, FHC) in the right amygdala (Horsley-Clarke coordinates: 22A-9L for monkey K.; 19A-9L for monkey H.; 17A-9L for monkey St.) based on preoperative structural MR images. During the same surgery, a low impedance (< 10 kΩ) reference electrode was inserted into the white matter of the frontal lobe. The fourth monkey (monkey Sh.) was implanted with a MRI-compatible recording chamber vertically above the left amygdala (Horsley-Clarke coordinates 22A and 9L) to allow recording neural signals with acute electrodes (which were the same type as the chronically implanted electrodes in the other monkeys, except the impedance at 1 kHz: 1 MΩ). To confirm the recording positions, glass capillaries were filled with a 2% copper sulfate solution and inserted into a recording grid at five different locations while a structural MRI (slice thickness 0.4 mm) was performed^22^. All surgical procedures were performed under strict aseptic conditions and monkeys were anesthetized with isoflurane (1%). Postoperatively, a T1- weighted anatomical MR image (0.4 mm isotropic resolution; TIM Trio, Siemens Healthcare) was performed to verify the anatomical location of the electrodes. Since comparing Horsley-Clark coordinates with the Paxinos atlas is unreliable because the coordinates change with age in monkeys, and since the distortion of the MRI signal by the electrodes could add to the inaccuracy, we could not precisely identify in which nucleus of the amygdala the electrodes were located. Based on the anatomical MRI, we estimate that the tip of the electrode was located in or near the basomedial amygdaloid nucleus in the three animals (Fig. 7a). Animal care and experimental procedures complied with the National, European, and National Institute of Health guidelines and were approved by the Ethical Committee of the KU Leuven.

### Stimuli

Ten classes of achromatic images—monkey and human bodies (excluding the head), monkey and human faces, four-legged mammals, birds, manmade objects (matched either to the aspect ratio of the monkey or the human bodies), fruit/vegetables and body-like sculptures—served as stimuli. Each class consisted of 20 images (from which one fourth is shown in Fig. 7b). The characteristics and the source of the stimuli are described in detail by Popivanov and colleagues^17^. Images were presented in a random order during passive fixation. If the monkey broke fixation, another random image from our 200 stimuli was shown. As described previously^17^, the human face stimuli were created from photographs being stored as part of the Tarrlab stimulus repository (courtesy of M. J. Tarr) and the NBU Faces Database, and were therefore unknown to the monkeys. The monkey face stimuli were all faces from three male monkeys from our colony, and therefore familiar to our monkeys.

Low-level image characteristics were equated across the different stimulus classes, such as mean luminance and aspect ratio. The mean aspect ratio of the monkey and human bodies differed since the upright human bodies tend to be more elongated than the monkey bodies. This was controlled for by using two classes of manmade objects—one matching the aspect ratio of the human bodies (object-H) and another matching the aspect ratio of the monkey bodies (object-M). The images were resized so that the average area per class was matched across all classes, except for the object-H and human bodies, but still allowing some variation in area (range 3.7°–6.7° (square root of the area)) within each class. This variation in size avoided potential clustering of the image classes based on local, pixel-based gray level differences. The mean vertical and horizontal extent of the images was 8.3° and 6.7° of visual angle, respectively.

Similar as in the experiment of Popivanov and colleagues^17,23^, the images were embedded into pink noise backgrounds having the same luminance as the images. Pink noise has a spatial frequency power spectrum similar to those of natural images. The noise background filled the entire display (height × width: 30° × 40° of visual angle). Each image was presented on top of 5 different backgrounds that were changed randomly during the experiment. Although unlikely, the use of different backgrounds may have (slightly) increased response variability.

### Electrophysiological recordings

The setup of the electrophysiological recordings was similar as in our previous studies (described in Popivanov et al.^23^). During the recordings, the monkeys sat in a primate chair, which was positioned directly in front of a screen at a distance of 86 cm. The gamma-corrected stimuli were projected onto the screen in front of the monkey. Stimuli were presented for 800 ms each with an interstimulus interval of approximately 500 ms during passive fixation. The pink noise background was present throughout the task, but refreshed together with the stimulus onset. Fixation was required in a period from 300 ms pre-stimulus to 800 ms post-stimulus within a square window of 2° × 2° of visual angle, after which the subjects received a juice reward. A trial was aborted when the monkey interrupted fixation in this interval. All 200 stimuli were presented foveally in a random order. Eye position was continuously tracked using an infrared video-based tracking system (EyeLink 1000, SR research Ontario). As in all our previous studies, the on- and offset of the stimulus was signaled by means of a photodiode detecting luminance changes in a small square in the corner of the display (but invisible to the animal), placed in the same frame as the stimulus events. A Digital Signal Processing-based computer system developed in-house controlled stimulus presentation, event timing, and juice delivery while sampling the photodiode signal, vertical and horizontal eye positions, LFP signals and behavioral events. For all subjects LFPs were amplified and filtered online using a 1–500 Hz bandpass filter (MCP, Alpha Omega Engineering) and saved for off-line analyses. In monkey K., monkey H. and monkey St., LFP signals were recorded through the chronically implanted electrodes. In monkey Sh., we recorded LFP signals with similar tungsten microelectrodes (impedance: 1 MΩ at 1 kHz; FHC) inserted through the dura by means of a 23-gauge stainless steel guide tube and a hydraulic Microdrive (FHC). Recording positions were chosen based on possible task-related single-unit activity in two grid positions. After recordings, we obtained an anatomical MRI with the electrode in the main recording position (Fig. 7a, bottom right). Simultaneously to the LFP recordings, we also obtained single unit and multi-unit activity. To that end, the neural activity was amplified and filtered between 500 and 5,000 Hz. Spike discrimination was performed online using a dual time window discriminator, and displayed with LabView and custom-built software.

### LFP analysis

All analyses were performed using custom-written Matlab (R2017b, Mathworks) programs, unless reported otherwise. LFPs were preprocessed by applying a digital 50 Hz notch filter (fourth-order Butterworth FIR filter; Fieldtrip Toolbox) to remove 50 Hz line contamination. Trials in which the signal exceeded the 5th–95th percentile window of the total amplitude input range were excluded from the analyses. As a quality check, we randomly split the datasets in half and repeated all analyses. If the results were not identical because of one trial with a large signal, we removed that trial. By convolving single-trial data using complex Morlet wavelets and taking the square of the convolution between wavelet and signal, we obtained the time-varying power of the signal for every frequency^24^. The complex Morlet wavelets had a constant center frequency—spectral bandwidth ratio (ƒ_0_/σ_ƒ_) of 7, with ƒ_0_ ranging from 1 to 150 Hz in steps of 1 Hz. Power was normalized per trial by dividing the power trace per frequency by the average power for this frequency in the 300 ms interval before stimulus onset. The data set was split in two and all analyses were repeated for both halves to check for consistency. We analyzed the LFP power in standard frequency bands: high-gamma (80–150 Hz), medium-gamma (50–80 Hz), low-gamma (25–50 Hz), beta (12–25 Hz) and alpha (8–12 Hz). LFP data were not corrected for average visually evoked potentials (VEPs), but removing the VEP yielded similar response patterns. We tested the LFP responses for significance in arbitrary time windows of [30–100 ms] to capture the fastest responses, [0–200 ms] and [200–500 ms] after stimulus onset. LFP power was averaged across trials to extract the average power per frequency over time. For the subjects implanted with chronic electrodes, we additionally analyzed the LFP power response per stimulus in the medium gamma band (50–80 Hz). The average LFP power response in this frequency band was ranked over categories and within categories based on the response in a 30 to 100 ms time window relative to stimulus onset in the odd trials. The even trials were then plotted according to this ranking. In a broader time window (0–200 ms after stimulus onset), we calculated the average gamma LFP response across categories for each recording position in each of the four monkeys to determine the time sample on which the LFP response peaked.

### Spike analysis

Custom-written scripts in Matlab (R2017b, Mathworks) were used for data analysis, as described before^25^. For each recording site, we assessed the visual responsiveness of the recorded neuron to the different stimulus categories by comparing the mean activity in a response window (0–300 ms after stimulus onset) to the mean activity in the baseline window (− 300 to 0 ms before stimulus onset, in which only a fixation point is presented to the subject) (t-test; p < 0.05). This yielded 20 responsive cells (10 in each grid position). We calculated net neural responses by subtraction of the mean multi-unit activity in the 0–800 ms interval after stimulus onset and the 300 ms interval before stimulus onset. For each cell, a random half of the trials were used for the ranking of the stimuli, while the other half were used to average the responses. Normalized activity was calculated with respect to the maximum responses across all 200 stimuli tested. The latency of category selectivity was determined as the first of three consecutive bins that showed a significant difference (t-test, p < 0.05) between the preferred and nonpreferred category.
